# Dosimetric verification of four dose calculation algorithms for spine stereotactic body radiotherapy

**DOI:** 10.1093/jrr/rrad086

**Published:** 2023-11-22

**Authors:** Hideaki Hirashima, Mitsuhiro Nakamura, Kiyonao Nakamura, Yukinori Matsuo, Takashi Mizowaki

**Affiliations:** Department of Radiation Oncology and Image-Applied Therapy, Graduate School of Medicine, Kyoto University, 54 Kawahara-cho, Shogoin, Sakyo-ku, Kyoto 606-8507, Japan; Department of Radiation Oncology and Image-Applied Therapy, Graduate School of Medicine, Kyoto University, 54 Kawahara-cho, Shogoin, Sakyo-ku, Kyoto 606-8507, Japan; Department of Advanced Medical Physics, Graduate School of Medicine, Kyoto University, 53 Kawahara-cho, Shogoin, Sakyo-ku, Kyoto 606-8507, Japan; Department of Radiation Oncology and Image-Applied Therapy, Graduate School of Medicine, Kyoto University, 54 Kawahara-cho, Shogoin, Sakyo-ku, Kyoto 606-8507, Japan; Department of Radiation Oncology and Image-Applied Therapy, Graduate School of Medicine, Kyoto University, 54 Kawahara-cho, Shogoin, Sakyo-ku, Kyoto 606-8507, Japan; Department of Radiation Oncology and Image-Applied Therapy, Graduate School of Medicine, Kyoto University, 54 Kawahara-cho, Shogoin, Sakyo-ku, Kyoto 606-8507, Japan

**Keywords:** dosimetric verification, dose calculation algorithm, spine SBRT

## Abstract

The applications of Type B [anisotropic analytical algorithm (AAA) and collapsed cone (CC)] and Type C [Acuros XB (AXB) and photon Monte Carlo (PMC)] dose calculation algorithms in spine stereotactic body radiotherapy (SBRT) were evaluated. Water- and bone-equivalent phantoms were combined to evaluate the percentage depth dose and dose profile. Subsequently, 48 consecutive patients with clinical spine SBRT plans were evaluated. All treatment plans were created using AXB in Eclipse. The prescription dose was 24 Gy in two fractions at a 10 MV FFF on TrueBeam. The doses were then recalculated with AAA, CC and PMC while maintaining the AXB-calculated monitor units and beam arrangement. The dose index values obtained using the four dose calculation algorithms were then compared. The AXB and PMC dose distributions agreed with the bone-equivalent phantom measurements (within ±2.0%); the AAA and CC values were higher than those in the bone-equivalent phantom region. For the spine SBRT plans, PMC, AAA and CC were overestimated compared with AXB in terms of the near minimum and maximum doses of the target and organ at risk, respectively; the mean dose difference was within 4.2%, which is equivalent with within 1 Gy. The phantom study showed that the results from AXB and PMC agreed with the measurements within ±2.0%. However, the mean dose difference ranged from 0.5 to 1 Gy in the spine SBRT planning study when the dose calculation algorithms changed. Users should incorporate a clinical introduction that includes an awareness of these differences.

## INTRODUCTION

Stereotactic body radiotherapy (SBRT) for the spine is gaining acceptance as the standard of care to improve local control outcomes and overall survival compared with conventional fraction radiotherapy [1–3]. This technique provides the benefit of escalating the radiation dose to the target volume while sparing the adjacent organs at risk (OARs) [4–12]. However, a dose calculation engine that can accurately account for the bone region needs to be developed to understand the radiation dose–response relationships for tumors and OARs.

The American Association of Physicists in Medicine Task Group 101 recommends the use of Type B and Type C dose calculation algorithms (superposition/convolution and Monte Carlo, respectively) for spine SBRT [6]. The behavior of radiation within the body varies due to differences in dose calculation algorithms. This phenomenon is particularly pronounced at interfaces, especially in the vicinity of metals [13, 14]. Even in Type C algorithms, which are considered the most accurate, dose consistency near metals is reported to be low [15–17]. In spine SBRT, the target is the bone, and although it is not as challenging as with metals, verifying dose at interfaces remains difficult but essential. In addition, the simulation of the radiation source and the collimating system is a great challenge, especially for spine SBRT treatments where field sizes can be very irregular (as small as single opened leaf pairs) and are fully dynamic both in terms of the leaf and beam angles, which require discretization of these degrees of freedom for analytical algorithms [18–21]. Zhen *et al*. [18] investigated the dose distribution of Acuros XB (AXB), anisotropic analytic algorithm (AAA) in Eclipse (Varian Medical Systems, Palo Alto, CA, USA) and collapsed cone (CC) convolution in Pinnacle (Philips Medical Systems, Andover, MA, USA) in the thoracic spine region. They reported that compared with AXB, the CC convolution algorithm yields a considerable overestimation of the target dose, whereas AAA underestimated the target dose with no statistical significance. Hardcastle *et al*. [19] assessed the dose difference between AAA and AXB and showed that the dose distribution in AAA was higher than that in AXB. Thus, different trends related to dose calculation algorithms have been reported in clinical cases; these trends can be attributed to differences in dose grids, dose-reporting modes, versions of treatment planning system (TPS) and limited treatment sites. Zhen *et al*. [18] used a rough dose grid (2.5 × 2.5 × 3.0 mm) and an older TPS version (Eclipse version 10.0). The dose indices are subject to change because of differences in dose calculation algorithms and conditions (dose grid and TPS version). A test on how the calculation algorithms behave in the presence of heterogeneities is therefore highly recommended in the quality assurance procedure of TPS. In particular, interface dosimetric measurements cannot be easily measured without a small volume detector and should be considered more important.

This study aims to evaluate dose differences between AXB and Photon Monte Carlo (PMC)/CC/AAA for spine SBRT in a simple geometry phantom and patient cases in conditions in which a fine dose grid and the latest TPS version were used.

## MATERIALS AND METHODS

### TPS and dose-calculation algorithms

The TPS used in this study included Eclipse (version 16.1, Varian Medical Systems, Palo Alto, CA, USA) and RayStation (version 10.0, RaySearch Laboratories AB, Stockholm, Sweden). The dose calculation algorithms in Eclipse were AXB and AAA (version 16.1) with heterogeneity correction. For AXB, the calculations were performed in a dose-to-medium dose-reporting mode, and material substances were allocated automatically based on a material table (version 13). The radiation dose calculation algorithms used for RayStation were PMC (version 1.3) and CC (version 5.3) with a heterogeneity correction. The computed tomography (CT) number to electron density table was used for AAA as the input, and the CT number to mass density table was used for AXB, CC and PMC [20, 21]. The spatial resolution of the dose grid was 1.0 mm for all dose-calculation algorithms.

### Dosimetric evaluation of four different dose calculation algorithms in homogeneous and heterogeneous phantoms

**Fig. 1:**
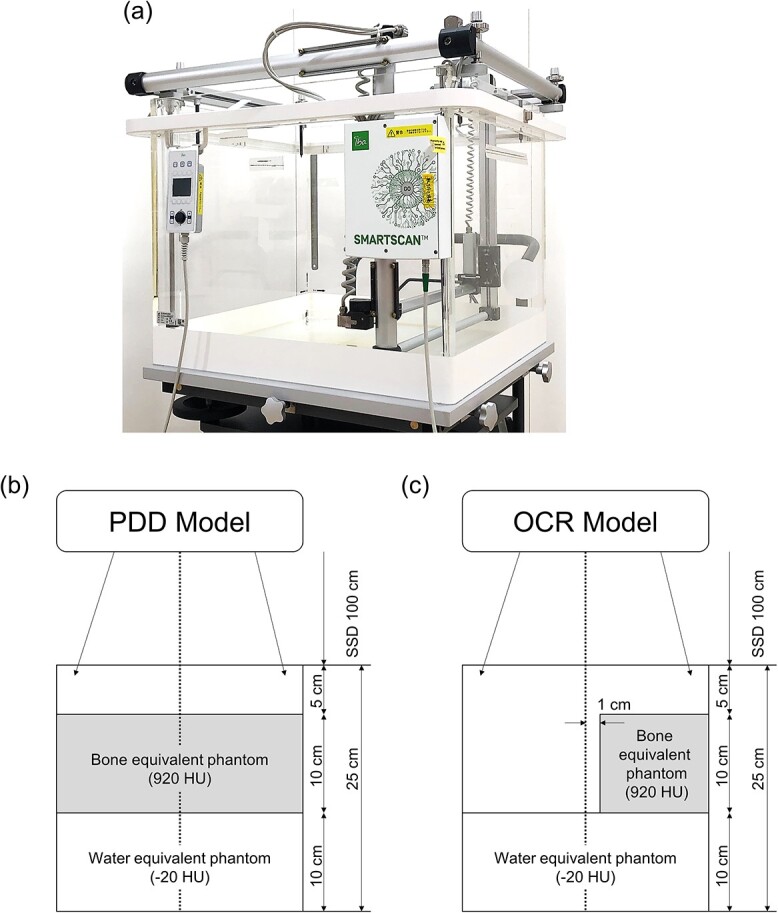
Geometries of (a) a 3D water phantom and (b, c) heterogeneous phantoms.

The percentage depth dose (PDD) and off-center axis ratio (OCR) in homogeneous and heterogeneous phantoms were determined. Figure 1 shows the experimental geometry of the homogeneous and heterogeneous phantoms. A 3D water phantom (Blue Phantom SMARTSCAN, IBA Dosimetry, Schwarzenbruck, Germany) was used as the homogeneous phantom to measure PDD and OCR (Fig. 1a). Regarding the heterogeneous phantom, water-equivalent phantoms [TM phantom; Taisei Medical, Inc., Japan, −20 Hounsfield units (HUs)] and bone-equivalent phantoms (RMI-450; GAMMEX, Inc., Middleton, WI, USA, 920 HU) were combined to evaluate the PDD and OCR (Fig. 1b and c). The assigned HU values are the averaged HU values for the CT images obtained by the respective phantoms.

TrueBeam (Varian Medical Systems, Palo Alto, CA, USA) was used with a 10 MV flattening filter-free (FFF) beam. The source-surface distance was 100 cm, and the field size was 10 × 10 cm [2]. For the PDD evaluation, the dose per monitor unit (MU) was used; measurements were performed using CC04 (IBA Dosimetry, Schwarzenbruck, Germany). For the OCR evaluations, the dose was normalized to the center position at a depth of 10 cm, and the measurements were conducted using CC04 in the 3D water phantom. Furthermore, a Gafchromic EBT3 film (Ashland Specialty Ingredients, Bridgewater, NJ, USA) was used in the heterogeneous phantom. The measurement values in the bone-equivalent phantom included multiple correction factors based on an earlier study [22].

### Patient, target and organ definition

Forty-eight patients who underwent spine SBRT at our institution between 2020 and 2022 were included in this study. This study was approved by our institutional review board and adhered to all relevant ethical tenets of the Declaration of Helsinki (approval number R1446). The details of the treatment sites are presented in Table 1. CT images (SOMATOM Definition, Siemens Medical Systems, Erlangen, Germany) were acquired with a 1 mm slice thickness. The gross tumor volume (GTV) was estimated based on contouring of planning CT and magnetic resonance imaging data. The clinical target volume was defined based on the International Spine Research Consortium consensus guideline [23]. A 2-mm margin was used for the planning target volume (PTV). The spinal cord and thecal sac were contoured based on the longitudinal relaxation (T1)-weighted MR image. A planning organ-at-risk volume (PRV) margin of 2 mm was added to the spinal cord (PRV_cord) but not to the thecal sac. PTVopt was defined as PTV minus PRV_cord or the thecal sac. Other organs were contoured based on the position of the spine.

**Table 1:** Details of the treatment site in spine SBRT

Site	No.
Cervical spine	9
Thoracic spine	23
Lumbar spine	10
Sacral vertebra	6
All	48

### Radiation treatment planning

All treatment plans were created using Eclipse for research purposes. The prescription dose (PD) was 24 Gy in two fractions with a 10 MV FFF photon energy on TrueBeam (Varian Medical Systems). PTVopt was prescribed based on the 90% PD with a volume that was at least 90% of the volume of the PTV, and the dose of the PRV_cord was <17 Gy. The dose constraints are listed in Table 2. AXB with a dose-to-medium ratio was used as the reference dose distribution. Thereafter, the dose calculation algorithm was changed from AXB to AAA, and the dose distributions were recalculated with the same MU. Treatment plans that were calculated using AXB and AAA were transferred from Eclipse to RayStation; the dose distributions were recalculated with CC and PMC while maintaining the AXB-calculated MUs and beam arrangement. The grid size for all dose calculation algorithms was set to 1 mm. The uncertainty of the dose calculation for the PMC was set to 1%.

**Table 2:** Dose constraint for spine SBRT plan

Structure	Metric		Optimal	Mandatory
GTV	D_98%_ [Gy]	>	24	22.8
	D_max_ [Gy]	<	35	
PTVopt	D_90%_ [Gy]	>	21.6	19.2
	D_max_ [Gy]	<	35	
				
PRV_cord	D_0.1cc_ [Gy]	<	17	19.3
Thecal sac	D_0.1cc_ [Gy]	<	17	19.3
Esophagus	D_0.1cc_ [Gy]	<	20	24
Trachea	D_0.1cc_ [Gy]	<	20	
Aorta	D_0.1cc_ [Gy]	<	40	
Peripheral nerve	D_0.1cc_ [Gy]	<	26	
Skin	D_max_ [Gy]	<	20	
Liver	D_mean_ [Gy]	<	8	
Kidney	D_mean_ [Gy]	<	4	11
Bowel	D_0.1cc_ [Gy]	<	20	

D_xx%_ = dose covering xx% volume of the region of the structure, D_mean_ = mean dose, D_yy cc_ = dose covering yy cc volume of the region of structure.

### Comparison of dose index values among four dose calculation algorithms and correlation between the HU value and the dose difference of GTV

Dose index values were extracted after the dose distributions were calculated. The evaluation dose indices included the dose coverage in 98% of the volume of the region of structure (D_98%_) and near maximum dose (D_2%_) for GTV and PTVopt; and the maximum dose (D_max_), D_0.03cc_ and D_0.1cc_ for the PRV_cord. The dose difference represents the local difference and is defined as


(1) \begin{equation*} \mathrm{Dose}\ \mathrm{difference}=\frac{{\mathrm{D}}_{\mathrm{eval}}-{\mathrm{D}}_{\mathrm{ref}}}{{\mathrm{D}}_{\mathrm{ref}}}\times 100\ \left(\%\right) \end{equation*}

where D_eval_ and D_ref_ represent the evaluation dose indices with PMC, AAA and CC, and the reference dose was calculated using AXB, respectively. The correlation coefficient between the mean HU value of the GTV and the dose difference of dosimetric indices (GTV D_98%_ and D_2%_) were calculated.

### Comparison of the gamma passing rate and dose difference view among four dose calculation algorithms

For 3D dose comparisons among the dose calculation algorithms, 3D gamma and dose difference view analyses were conducted using the gamma function from the Python module PyMedPhys [24]. The evaluation in the 3D gamma analysis used the acceptability criteria of a 2% dose difference and 2 mm distance-to-agreement (2%/2 mm) and 1%/1 mm with a threshold dose of 10% [18, 25, 26]. The reference dose distribution was AXB when the gamma index and dose difference were calculated.

### Statistical analyses

Statistical analyses were performed by EZR [27]. The dose difference among the four dose calculation algorithms was evaluated using analysis of variance (ANOVA). After the ANOVA of the dose-volumetric data for the calculation algorithms, the data were statistically analyzed using the Bonferroni method. Correlations between parameters were determined using Pearson’s linear correlation coefficient; the statistical significance was set at *P* < 0.05.

## RESULTS

### Dosimetric evaluation for different dose calculation algorithms in homogeneous and heterogeneous phantoms

**Fig. 2:**
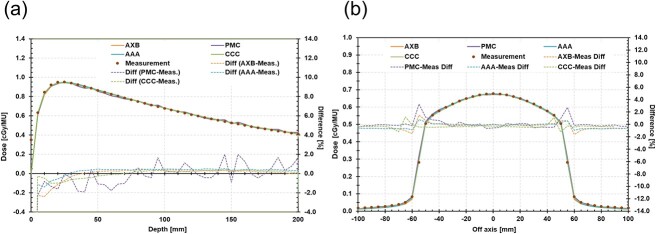
Absolute depth dose (a) and OCR (b) in homogeneous phantom with a 10 × 10 cm^2^ field size. OCR is profile at 10 cm.

**Fig. 3:**
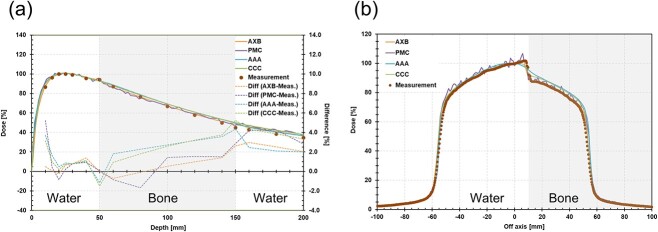
PDD (a) and OCR (b) in heterogeneous phantom with a 10 × 10 cm^2^ field size. OCR is profile at 10 cm.

In a homogeneous phantom, the mean dose difference between the calculated and measured PDD and OCR was within 1% for all dose calculation algorithms (Fig. 2a and b). In the heterogeneous phantom, the results from AXB and PMC agreed with the measurements within ±2.0%, whereas AAA and CC appeared to show increases of up to 5.5% (Fig. 3a and b).

### Dose index values calculated by four dose calculation algorithms

**Fig. 4:**
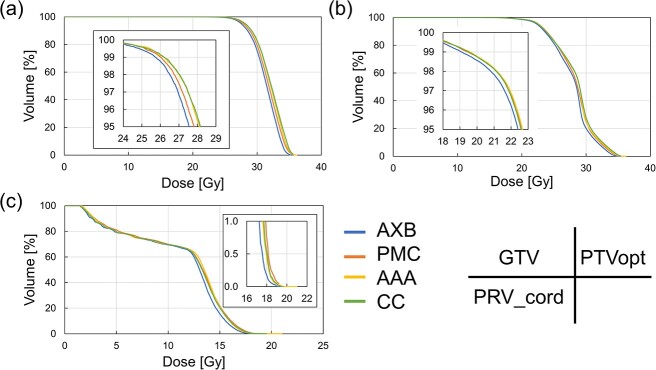
DVH in the representative patient for (a) GTV, (b) PTVopt and (c) PRV_cord.

**Fig. 5:**
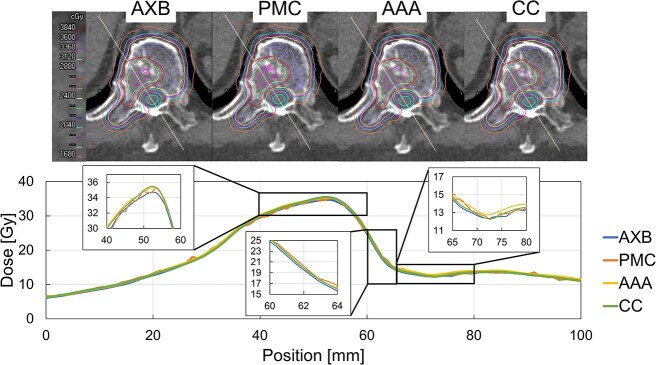
Dose distribution and line dose profile in a representative patient case calculated by AXB, PMC, AAA and CC.

The dose index values calculated using the four dose calculation algorithms are listed in Table 3. The mean ± standard deviation (SD) of the PTVopt and GTV in AXB were 20.2 ± 1.3 Gy and 25.0 ± 3.1 Gy for D_98%_, and 33.8 ± 0.9 Gy and 35.0 ± 0.5 Gy for D_2%_. The dose indices in the PRV_cord were 19.9 ± 0.8 Gy for D_max_, 17.7 ± 0.6 Gy for D_0.03cc_ and 17.0 ± 0.6 Gy for D_0.1cc_. Figure 4 shows the dose–volume histogram (DVH) of a representative patient. The dose index values in three algorithms (PMC, AAA and CC) were higher than those in AXB in all organs. Figure 5 shows the dose distribution and dose profile line calculated using the four dose calculation algorithms.

**Table 3:** Dosimetric index value and dose difference for GTV, PTVopt and PRV_cord calculated by AXB, PMC, AAA and CC

		Mean ± SD [Gy]			
Structure	Index	AXB	PMC	AAA	CC
GTV	D_98%_	25.04 ± 3.09	25.35 ± 2.98 [1.36 ± 1.22%]	25.66 ± 3.16 [1.80 ± 0.97%]	25.75 ± 3.16 [2.05 ± 1.13%]
	D_2%_	34.97 ± 0.52	35.55 ± 0.51 [1.64 ± 0.72%]^*^	35.57 ± 0.66 [1.70 ± 1.07%]^*^	35.86 ± 0.66 [2.52 ± 1.17%]^*^
PTVopt	D_98%_	20.23 ± 1.29	20.47 ± 1.32 [1.18 ± 0.80%]	20.59 ± 1.34 [1.79 ± 0.88%]	20.56 ± 1.31 [1.66 ± 1.02%]
	D_2%_	33.81 ± 0.85	34.22 ± 0.89 [1.22 ± 0.66%]	34.40 ± 0.84 [1.77 ± 0.85%]^*^	34.67 ± 0.84 [2.56 ± 0.95%]^*^
PRV_cord	D_max_	19.88 ± 0.76	20.70 ± 0.88 [4.12 ± 2.05%]^*^	20.12 ± 0.68 [1.25 ± 2.07%]	20.39 ± 0.79 [2.58 ± 1.64%]^*^
	D_0.03 cc_	17.68 ± 0.60	18.25 ± 066 [3.24 ± 0.87%]^*^	17.89 ± 0.56 [1.24 ± 1.1%]	18.08 ± 0.65 [2.27 ± 1.13%]^*^
	D_0.1 cc_	17.02 ± 0.62	17.54 ± 0.62 [3.07 ± 0.83%]^*^	17.24 ± 0.60 [1.32 ± 0.98%]	17.38 ± 0.64 [2.13 ± 1.10%]

Note: Values with an asterisk represent a statistically significant difference (*P* < 0.05). The values are shown as the mean ± SD dose value and the relative dose difference value in parentheses.

AAA = anisotropic analytical algorithm, D_xx%_ = dose covering xx% of the structure volume.

### Comparison of the dose index values among four dose calculation algorithms

Table 3 summarizes the dose differences among PMC-AXB, AAA-AXB and CC-AXB. The mean dose difference was within 4.2% (0.82 Gy) for all dose calculation algorithms. The mean dose value of AAA yielded a slightly higher value [1.8% (0.63 Gy)]; no significant differences were observed compared with AXB (this excludes D_2%_ for GTV and PTVopt). The mean dose values of PMC and CC were within 4.2 (0.82 Gy) and 2.6% (0.51 Gy) higher than that of AXB for all dose indices. D_98%_ for GTV and PTV opt, and D_max_ and D_0.03cc_ for PRV_cord of CC yielded significantly higher values than that of AXB (*P* < 0.05). The D_98%_ values for GTV and PRV_cord of all dose indices for PMC yielded significantly higher values than that of AXB (*P* < 0.05).

### Correlation coefficient between the mean HU value of GTV and dosimetric indices

**Fig. 6:**
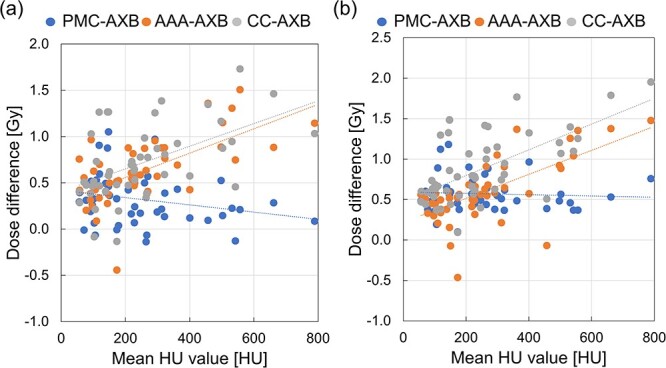
Correlation between the dose difference at (a) GTV D_98%_ and (b) D_2%_ and the GTV mean HU value. Blue, orange and gray circles indicate the dose differences of PMC, AAA and CC, respectively, as referenced by the AXB dose.

Figure 6 shows the mean HU values and different dosimetric indices of the GTV. The correlation coefficient value between the mean HU value and D_98%_ and D_2%_ of GTV was −0.24 (*P* = 0.10) and − 0.05 (*P* = 0.71) for PMC-AXB, 0.65 (*P* < 0.05) and 0.66 (*P* < 0.05) for AAA-AXB and 0.52 (*P* < 0.05) and 0.65 (*P* < 0.05) for CC-AXB, respectively.

### Comparison of the gamma passing rates and dose difference views among the four dose calculation algorithms

**Fig. 7:**
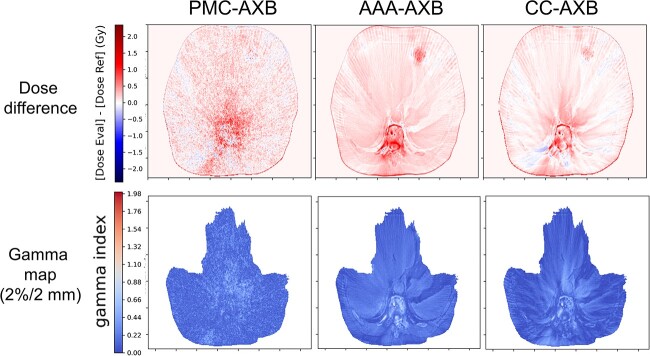
Dose difference and gamma maps (2%/2 mm) in the representative patient case for PMC-AXB, AAA-AXB and CC-AXB.

The spatial distribution of the gamma index (2%/2 mm) and dose difference is presented in Fig. 7 The dose distribution of AAA was higher than that of AXB in the entire body; in contrast, the dose distributions of PMC and CC were lower than that of AXB in the entire body. Table 4 presents the results for the gamma indices. The mean ± SD values of the gamma 2%/2 mm and 1%/1 mm were 99.9 ± 0.2% and 98.8 ± 1.6%, respectively.

**Table 4:** Result of 3D gamma passing rate between PMC/AAA/CC and AXB

	Mean ± SD [%]		
Gamma passing rate	PMC-AXB	AAA-AXB	CC-AXB
2%/2 mm	99.99 ± 0.17	99.84 ± 0.19	99.80 ± 0.18
			
1%//1 mm	99.62 ± 0.29	98.19 ± 1.39	98.60 ± 0.59

AAA = anisotropic analytical algorithm.

## DISCUSSION

An accurate understanding of dose distributions in the SBRT treatment planning for spine metastases is required because of the steep dose distribution and relatively higher HU values in the bone region than in soft tissues. Our results for simple geometries were comparable to those of other researchers [20, 21]. From the results of the patient cases, calculations using PMC, AAA and CC yielded calculated values that were higher than those calculated using AXB. Although Zhen *et al*. showed opposite results [18], some researchers reported that the AAA dose was overestimated in comparison with MC simulations in bone regions [19, 20, 25]. Feygelman *et al*. [28] and Lee *et al*. [29] indicated that the CC dose overestimated the 3% dose in the bone region compared with the PMC dose. Therefore, Type B (superposition/convolution) algorithms would overestimate dose distributions more than Type C algorithms, as indicated in previously published studies. Although (to our knowledge) no other report has directly compared AXB and PMC in the bone region, our findings showed that the dose profile between AXB and PMC was within 2% in the bone-equivalent region. Our results showed that the PMC outcomes were slightly overestimated in comparison with those obtained with AXB. We assume that the significant difference in the PMC values compared with those of AXB can be attributed to differences in MLC modeling and algorithms. For small beams used in SBRT, the beam penumbra becomes larger because of the lateral electron transport in the medium at higher beam energies [6]. Thus, MLC parameters were critical in creating dose distributions in spine SBRT. Regarding the MLC parameters, Eclipse allows the input of the transmission and dosimetric leaf gap, whereas RayStation additionally allows the input of the tongue and groove values. Therefore, it is possible that differences between Eclipse and RayStation were observed in the spine SBRT. In the case of VMAT, where MLCs move in a complex manner, differences in dose distribution occur among TPS. Moreover, AXB utilizes the linear Boltzmann equation solvers to calculate the particle fluence. It produces results without statistical fluctuations but may introduce systematic uncertainties. PMC uses random numbers to calculate particle fluences. Statistical uncertainty exists owing to the number of particles. Therefore, the dose difference between AXB and PMC is considered to be caused by statistical uncertainty. According to a previous report, dose differences of at least 5–10% were detectable in the bone region [19–21, 25,28–32]. Furthermore, Hoffman *et al*. reported the comparison between AXB and SciMoCa based on the Monte Carlo algorithm [33]. These authors indicated that Acuros yielded a slightly higher dose than SciMoCa in bone regions [up to 3% (1.5 Gy)]. Although AXB yielded a slightly higher dose compared with that yielded by Monte Carlo, the agreement of the outcomes was extremely good [33]. All differences among the dose calculation algorithms investigated in our study were within these criteria.

The PRV of the spinal cord dose is essential for spine SBRT. If the dose differences between different dose calculation algorithms are large, it may be necessary to modify dose constraints. The comparison of the maximum doses (D_max_, D_0.03cc_ and D_0.1cc_) suggests that the dose difference of point doses, in particular D_max_, is easily affected by the difference in dose calculation algorithms. The dose difference in PRV_cord tends to be larger for the Type C algorithm in particular because it is easily affected by statistical uncertainty.

In bone regions, the dose error increased at increasing HU values, as reported in the previous literature [19]. Hardcastle *et al*. reported a significant correlation between the difference in AAA-AXB and the mean HU value of GTV [19]. The effect of the difference in AAA-AXB and CC-AXB was similar; however, PMC-AXB showed no correlation. The variation caused by changes in the HU values is expected to be small because the PMC is an equivalent algorithm to AXB. The dose difference in the Type B algorithm can increase as a function of the HU value range compared with the Type C algorithm. AAA and CC are calculated using dose-to-water, while AXB and PMC are calculated using dose-to-medium [29]. Differences in the method that take into account the material density will likely lead to systematic errors with increases in the HU values as different calculation methods produce different results.

Our detailed 3D comparisons between the AXB and the other algorithms provided valuable insights. The dose difference was visually apparent in the bone region (Fig. 5); this indicates that the differences were attributed to the dose calculation algorithms or TPS. The gamma pass rate was more than 99% for 2%/2 mm, which is in close agreement with the results of previous research [18, 25, 26]. Even at the more stringent gamma criteria of 1%/1 mm, the mean value was >98%; this indicates that the dose difference is attributed to the dose difference within the tissue rather than dose expansion and contraction because of intertissue effects.

This study is associated with the following limitations:

- We did not evaluate the dose distribution between the calculated and measured values in the spine phantom. Some studies indicated the accuracy of the dose distribution between the calculated and measured values using the spine phantom [28, 30]. Calculation- and measurement-based comparisons are required.
- We used one statistical uncertainty value in the PMC to evaluate dose indices. The change in statistical uncertainty from 0.5 to 1.0% increased by 1–2% of the dose value [28], although a statistical uncertainty of 1% was selected from the viewpoint of calculation time in our study.
- We only applied a 10 MV FFF beam. The stopping-power ratio depends on the energy; the correction factors for the bone region in the measurement value were changed [22]. The differences in the energy types were not assessed.

## CONCLUSION

This study evaluated dose differences between AXB and PMC/CC/AAA for spine SBRT in a simple geometry phantom and patient cases, utilizing a fine dose grid and the latest TPS version. The phantom study showed that the results from AXB and PMC agreed with the measurements within ±2.0%. However, the mean dose difference ranged from 0.5 to 1 Gy in the spine SBRT planning study when the dose calculation algorithms changed. Users should incorporate a clinical introduction that includes an awareness of these differences.

## Data Availability

The data are available on request from the authors.
